# Not All that Is Solid Melts into Air? Care-Experienced Young People, Friendship and Relationships in the ‘Digital Age’

**DOI:** 10.1093/bjsw/bcu152

**Published:** 2015-02-05

**Authors:** Robin Sen

**Affiliations:** University of Sheffield, Elmfield, Northumberland Road, Sheffield, S10 2TU, UK

**Keywords:** Looked after children, the internet, social media

## Abstract

The circumstances of those who are, or have been, in the care system may augment concern about their use of mobile phones and the internet, but little is specifically known about such use. Presenting findings from an exploratory study which investigated the experiences and views of six care leavers and four looked after children, this paper considers their social contact via mobile phones and the internet. Exploration of the study data is located alongside wider empirical findings around internet use and critical consideration of theoretical insights from the work of Bauman, Castells and LaMendola. Participants' reported use of digital media was not substantially different to that of their peer group: their core virtual networks had significant overlap with their core offline networks and social contact via digital media could provide welcome, if limited and individualised, social support. The most prominent difficulty arising from the use of these media was forms of verbal abuse by those known to the young people offline. While the centrality of digital technology within young people's lives influenced the way they communicated, underlying issues within their social relationships reflected greater similarity with a pre-digital age than has sometimes been suggested.

## Introduction

Discourse regarding young people's use of digital media is often focused on the dangers it poses. In August 2013, concerns were re-ignited by the suicide of British teenager Hannah Smith following abuse she received on the social networking site Ask.fm. David Cameron responded by declaring that social networking sites which do not address online bullying should be boycotted (BBC, 2013). While the case provided a stark reminder of the potential risks involved in social media use, it has been argued that undue focus on ‘extreme and exceptional cases’ such as this has created a moral panic about young people's internet use (Ballantyne *et al*., 2010, p. 96). Mainstream media coverage of the impact of young people's use of digital media on their social relationships has also centred on negatives. Livingstone (2008) and Livingstone and Brake (2010) list media stories which, amongst other things, decry young people's lack of sense of privacy online, the self-referential and trivial content of online communication and the undermining of friendship via social networking sites. A more recent newspaper article reported that, despite their large numbers of online friends, young people are ‘lonely’ and ‘socially isolated’ (Hartley-Parkinson, 2011). While acknowledging the sensationalism in such coverage, Livingstone (2009) has argued that approaches to young people's use of the internet need to balance ‘risks’ and ‘opportunities’ and that research should seek to more clearly establish what those are. She has also argued academic research has preferred to focus ‘on the positives and examine online opportunities’ (2009, p. 152), rather than investigating potential risks.

By contrast, the empirical research on young people's use of the internet within the social work field is sparse, and has focused on how best to mitigate online risks (Fursland, 2010, 2011; May-Chahal *et al*., 2012). This has a rationale as the dangers posed via new technology are more likely to be evident in the lives of young people receiving social work support. For example, evidence regarding child sexual exploitation in groups and gangs indicate this as an issue of significant concern in which new technology plays a role (Beckett *et al*., 2013; Berelowitz *et al*., 2013; CEOP, 2013). Victimisation often occurs both online and offline, and the process of exploitation can be initiated through online contact and grooming. The experience of sexual exploitation is a gendered one whereby the vast majority of victims are girls and young women and the perpetrators male. Young people with experience of the care system are also notably over-represented in current data regarding child sexual exploitation (OCC, 2012; CEOP, 2013). Research also suggests that young people who have experienced prior abuse offline are more susceptible to online grooming (May-Chahal *et al*., 2012) and there is considerable professional anxiety about unmediated contact between looked after children and adopted children and their birth families via new technology (Fursland, 2010, 2011; Sen, 2010).

Responses require careful consideration, however. The exact relationship between online and offline vulnerability still needs to be better understood (Livingstone and Palmer, 2012) and the evidence does not support an assumption that young people with care experience are, per se, at greater risk online. Even where there is greater concern about a young person's safety, recognition is needed that their online activities will present a complex mixture of risks and opportunities over which they will exert their own judgement and agency.

Further understanding of this issue depends on greater insight into the online experiences of young people receiving social work support. This paper contributes to the knowledge base by reporting findings from a study exploring the perspectives of six care leavers and four looked after children regarding commonly discussed risks associated with digital media and their own use of such media. The paper focuses on participants' experiences of using digital media for social contact.

## Theorising digital relations

Concerns about the impact of digital technology on young people's social relationships resonate with pessimistic theories of individualisation in late modernity. It has been argued that the dissolution of traditional civic, community and social bonds arising from globalisation leads to human relationships which are more fragile and superficial (Beck, 1992; Bauman, 2000). For Bauman (2000), life under conditions of liquid modernity is characterised by feelings of ‘precariousness, instability and vulnerability’ (p. 160). While he is not a theorist of the ‘digital age’ as such, Bauman's observations are frequently illustrated with examples from, or clearly applicable to, it. In respect of internet dating sites, he comments that ‘unlike old-fashioned relationships virtual relations seem to be made to the measure of a liquid modern life setting …, “virtual relationships” are easy to enter and exit’ (Bauman, 2003, p. xii). His observation that our times have seen the redefinition of the boundaries between the public and the private, such that ‘private dramas are staged, put on display, and publically watched’ (2000, p. 70), is a broader social comment, but resonates with concerns about privacy and self-disclosure on the internet, particularly amongst young people. Bauman (2003, 2005) also critically traces the impact of digital technology on the character of human communication, arguing that it has become less about the transmission of meaning than the fact of being connected: ‘We belong to talking, not what is talked *about* … the union only goes so far as the dialling, talking, messaging. Stop talking and you are out. Silence equals exclusion’ (Bauman, 2003, pp. 34–5, emphasis in original).

Of core relevance to the debate around relational depth and digital technology is the ability to connect with those who are physically distant. For Castells (2001), this leads to a ‘space of flows’ rather than ‘a space of places’. This enables participation in physically remote ‘communities of choice’ where relationships are not limited by place (Castells, 2003). For Bauman (2000), however, the rise of ‘virtual proximity’ to the detriment of ‘physical proximity’ not only means that we are more distant from those physically around us, but ‘renders human connections simultaneously more frequent and more shallow, more intense and more brief’ (2003, p. 62). LaMendola (2010) brings the debate into social work practice, drawing on Levinas (1969). He considers whether psychological and emotional contact which emerges from trying to ‘know the other’ in face-to-face engagement is extended by new technology and argues that digital technology means such contact is no longer limited to physical co-presence. Following Rettie (2009, in LaMendola, 2010), he distinguishes between digitally mediated communication which allows intersubjective engagement—typically synchronous communication such as video links—and asynchronous communication such as text and e-mail which do not.

## Young people's online connections

Research around adult internet use has found online social engagement tends to be more individualised and less reciprocal than offline community participation and represents ‘networked individualism’ rather than engagement in online ‘communities’ (Wellman, 2001). Reich's (2010) study found networked individualism also described young people's online social networks. These networks tended to lack some of the defining features of a community such as a sense of belonging and identification, influence on the community and investment by the community, although they did facilitate communication and could support the existence of offline networks through this. A consistent finding is that young people mostly communicate online with those they already know offline and the content of most communication tends to be about everyday issues (Gross, 2004; boyd, 2008; Subrahmanyam *et al*., 2008; Reich *et al*., 2012). The effect of online social connection is less clear. Attewell *et al*. (2003) found some substitution effects, with adolescents who had a home computer spending less time playing outside. Gross (2004), however, found no association between young people's internet use and well-being while Valkenburg and Peter (2007) found pre-adolescents and adolescents who spent time online with existing friends were more likely to feel closer to these friends.

Online experiences will, however, be socially mediated and can vary. A study of ‘sexting’ amongst teenagers in mainstream London schools (Ringrose *et al*., 2012) highlighted how new technology has ‘amplified’ peer-to-peer sexual pressure in youth relationships, particularly for girls. A commonality between this research and that on sexual exploitation (Beckett *et al.*, 2013; Berelowitz *et al*., 2013) is the gendered nature of experience. Young people's accounts indicated that the sexual objectification of girls and young women worked alongside long-standing social constructions of sexual activity as a highly positive sign of status for boys and young men and a highly negative one for girls and young women.

Guzzetti's (2006) small-scale in-depth observational study of two young women's online interaction provides a counterpoint. It illustrates how the women furthered their interest in punk rock music and explored aspects of identity through online media such as message boards and zines. After analysing the young women's discursive online interaction, Guzzetti concludes that ‘the online environment may provide safe spaces for girls that are not found offline’ (p. 158). There will be limits to how far online interaction is insulated from wider social constructions though. In considering the potential for online media to create ‘female counter-publics’, Salter (2013) notes that any counter-hegemonic discourse will be resisted as it tries to spread. While online interaction provides a potentially global platform for counter-discourse, it is not without its own constraints.

Generalisations regarding young people's experience of new technology can provide helpful insights therefore, but empirical evidence also suggests some variation. The importance of remaining open to the plurality and individuality of young people's experience of new technology, while locating broader social constructions it operates within, is emphasised.

## Care-experienced young people and online social support

As there may be greater risks for looked after children and care leavers online, there may also be greater opportunities. The social isolation faced by care leavers is well documented (Stein, 2012) as is the importance of social support in helping young people overcome adverse life situations (Gilligan, 2000). While the care system can provide continuity of care, multiple placement moves can fracture relationships and networks for young people in long-term care (Boddy, 2013). Online interaction is not a substitute for enduring caring relationships but it can help sustain social contact and can galvanise and deepen social support (Valkenburg and Peter, 2007).

Structural limits to the social support an individual can garner through online activity will exist. Technical knowledge, skills and online access will condition a young person's ability to take advantage of online opportunities. And, if young people's online social networks principally comprise offline networks, the same limitations to the quality of social support they offer will apply. Nevertheless, young people can deepen relationships by connecting online and online communication can help facilitate offline group membership (Reich, 2010) which can provide access to extended social networks and greater social support. Therefore, it is proposed that a situation of ‘bounded agency’ is likely to exist in respect of the social support those in or exiting the care system can garner through online interaction. Furlong (2009, p. 353) has defined this perspective in respect of youth transitions as one which recognises the importance of context in shaping experience and resources in influencing outcomes but which also recognises that ‘young people themselves have always attempted to influence outcomes, realise their aspirations and move forward reflexive life projects’.

## The study

Data were collected in 2011 and consisted of two interviews with ten participants. One care leaver was unavailable for a second interview so nineteen interviews were completed. Use of digital media was defined as any use of a mobile phone or the internet for any purpose. The first interview was structured around four vignettes concerning a potential sexting scenario, a request from a friend of a friend on a social networking site, a contact request from an absent parent to a child in foster-care and a ‘cyber-bullying’ scenario. The second, more unstructured, interview explored everyday usage based around a daily log the young person had kept about their mobile and internet use over a previous week.

The sample was purposive, consisting of six recent care leavers and four looked after young people recruited through two organisations in the same town. Four participants were female and six male: the gender of each participant is reflected by the choice of pseudonym in Table 1. Two of the participants had moderate learning difficulties and one Asperger syndrome. Eight of the participants were white British and two mixed white/Asian. All the participants were, or had been, in long-term foster or residential placements.

**Table 1:** Participant details

Participant pseudonym	Looked after status, age
Diane	Looked after child, 13
Geoff	Looked after child, 13
Oliver	Looked after child, 14
Tanya	Looked after child, 15
Adam	Care leaver, 18
Donna	Care leaver, 19
Graham	Care leaver, 19
Nick	Care leaver, 19
Tracey	Care leaver, 19
Harry	Care leaver, 21

Interviews were recorded and transcribed. The focus of this paper is unstructured data from the first interviews and data from the second interviews which were analysed by a process of qualitative analysis outlined by Miles and Huberman (1994) and influenced by the process of template analysis described by King (1998). The final template grouped data under the themes of ‘Platforms and technology used’, ‘Frequency and duration of use’, ‘Purposes of use’, ‘“Likes” of use’, ‘“Dislikes” of use’, ‘Personal circumstances and use’, ‘Online interaction with those known offline’ and ‘Online interaction with those unknown offline’. The use of Nvivo 9 assisted in the analysis.

Participants were from the same geographical area and were recruited via two organisations which organised drop-in services for looked after children and care leavers, respectively. Attempts were made to gain a sample that had some balance in terms of age, gender, disability and ethnicity. The four looked after children, on the one hand, and the six care leavers, on the other, knew each other from the drop-in via which they were recruited and shared some networks. A greater degree of overlap in experience than in a more diverse sample is therefore likely. Participants were all also young people who were accessing formal support services. The experiences of other care-experienced young people who are not accessing supports in this way may be substantially different. Interviews were conducted by the author, someone previously unknown to participants. This may mean that participants were less likely to admit to experiences or behaviour by which they were embarrassed or viewed as intimate.

Ethical approval was granted by the University of Sheffield with subsequent approval granted by the relevant local authority of the four looked after children and the two organisations through whom the young people were recruited. Young people indicated a verbal willingness to take part in the study prior to first interview and written consent was provided before each interview. The possibility that the interviewer would need to pass on information where safeguarding issues were identified was discussed with participants prior to their giving consent. Interviews were conducted in private spaces within the drop-in centres such that staff who knew the young people were available should a participant become distressed.

## Means and forms of social contact via digital media

All participants except Nick had access to their own laptop or desktop computer at home and this was the principal means of going online. Mobiles were also used for texting and to connect to the internet but making calls on them was interestingly rarer. Facebook was the primary social networking platform which participants used: all had an account and nine accessed it at least daily. For three of the four looked after children, this was the only social networking platform they used, although Tanya also used deviantARt, a platform for uploading and commenting on artwork where there is some opportunity to interact with others. Four of the six care leavers regularly also used other platforms which had been popular before pre-eminence of Facebook—Bebo and ‘MSN’ (Windows Messenger, formerly MSN Messenger, which was operational at the time of data collection but is now defunct).

The ubiquity of Facebook was however a disadvantage for Nick, who stated its popularity had led him to start looking for alternative platforms:

I don't like to be like everybody else, I like to show individuality, this is me, I am not this person, I am somebody else.

boyd (2008) has illustrated how self-expression on social networking sites can be central to young people's identity. Nick's comments suggest that identity could be attached to the platform a young person uses, as well as the content they have on it, and notably pre-figured Facebook's own concern that, due to its ubiquity, younger users were migrating to alternative social media platforms (Facebook, 2013).

Young people's accounts of their connectivity were consistent with ‘networked individualism’ (Wellman, 2001). Connecting with others online, particularly by mobiles, frequently occurred when other people were physically co-present. However, online engagement tended to be individualised rather than shared with those who were physically there. The exceptions were watching video clips or film or television episodes via digital media but these shared activities rarely involved online communication.

All four looked after children had smart phones when first interviewed, while only one care leaver did. Financial resources are needed to keep pace with rapid technological change and none of the care leavers was in full-time employment. Some of the care leavers' comments indicated they were conscious of falling behind and demonstrated obsolescence—even though the mobiles they had were functional, they were lowly valued:

I've got one of those piece of rubbish phones that's from back in 2009 (Harry).

Well I did [have an internet-enabled mobile] but I got my phone stolen, so now I am stuck with a little crappy thing (Donna).

Being without the latest technology could affect connectivity. The longest periods the looked after children had been without online connection were due to either choice or holidays abroad. For five care leavers, it was due to computers or mobiles breaking down, mobiles getting lost or being stolen, being unable to afford internet access or practical barriers: Nick, for example, reported that Wi-Fi was not permitted in the hostel where he was staying so he had to connect via his mobile, the connection speed of which could be slow. Paradoxically, care leavers also tended to spend significantly longer online. The looked after children spent between thirty minutes and two hours online for social purposes each day, with longer at weekends, although all reported regularly checking for Facebook updates at school by mobile. Five of the care leavers spent more than four hours a day online, with Harry reporting a maximum of eight hours per day and Adam regularly spending ‘a good ten hours’ online including time undertaking a range of practical, educational and social activities.

## Online networks

The seven respondents who recalled had a mean number of 107 Facebook Friends, ranging between fifty-seven and 323. This compares to a mean of 176 friends amongst US students aged thirteen to nineteen in the study of Reich *et al*. (2012). Young people's Facebook Friends were principally those they had met offline and, for six of the young people (the four looked after children plus two of the care leavers), the great majority of Facebook Friends were known to them offline first. For two looked after children, a birth parent and other adult birth family members were amongst the Friends and, for one other looked after child, it included a birth sibling in a separate placement, as well as her foster-carer. While the six participants all had some online contact with people not known to them offline, this was either fleeting—for example, Geoff described playing Xbox games online against ‘random people’ where any interaction was limited to playing against others in a given one-off game—or through trusted offline sources—for example, Tanya had a Facebook Friend abroad who was the child of a friend of her foster-carer.

That online networks and offline networks were largely the same was emphasised by Nick's comments about Skype:

… the Skype thing it sounds like a great idea but who I am I going to Skype, all of my people live very close, I don't really need to Skype them so why are they putting that on to me as well? I don't need that extra option.

For him, the connectivity of a ‘space of flows’ offered via Skype appeared an irritation, rather than a liberation, precisely because his important networks were tied to locality.

All participants interacted regularly online with smaller numbers of Facebook Friends within their larger networks, thus a core virtual network existed like a core offline social network. The key advantages of this type of communication were that it was ‘quicker and easier’ (Geoff) and that it allowed ‘free communication between people’ (Adam). It was also clear that this type of contact was highly valued:

I need to use it regular, need to stay in touch with people. I need to stay in touch with people and know what they are doing and that. My family (Oliver).

… the Internet it's like a big part of my social life is there because usually when I switch the computer on it's like right MSN, check my emails, Facebook to see what's going on (Adam).

### ‘Private and like all about me’

Ballantyne *et al*. (2010) argue that, contrary to popular representation, young people tend to be very protective of their online privacy, although their conception of what is private may differ from older generations. Participants' accounts suggested this was true of them. All but one, who was unsure, reported that their Facebook profiles were not publically viewable, though there was frequent confusion over whether profiles were limited to Facebook Friends or wider networks.

Donna had profiles on both ‘MSN’ and Facebook and had different criteria for accepting contacts and posting information according to the platform she was using:

I use them in different ways, like Facebook it's mainly for my friends that actually know me but MSN doesn't hold any information about me apart from my e-mail address, like some people they do try to add me on Facebook but I just block them because my Facebook is more private and like all about me.

In one of the few suggestions that care experience influenced participants' use of digital media, Donna also remarked she was careful of what detail she posted about her whereabouts on her status updates because:

… my foster parents are right like safety aware and they tell me not to put stuff like that on Facebook and plus it's got nothing to do with anybody where I am.

Oliver commented that an advantage of his online communication was that ‘when it's face to face it's normally at school or here [the drop-in] and there is no privacy’. As well as individually messaging friends on Facebook, he also regularly described using wall posts and messaging on Facebook to multiple friends at the same time, so that, by privacy, he appeared to mean an absence of offline adult supervision.

Participants' sense of privacy was also suggested by their unease with the facility to be ‘tagged’ in photos on Facebook without giving express permission. Nick's comment was typical:

… if you're in the photo you can [be] tagged and then you're all over Google. I don't like that, they should make you sign up to it first.

Adam shared this concern but also raised the question of ‘ownership’ of the photo once posted:

… say we were friends on Facebook—I could own a photo, tag you in the photo, yet you could then share it to someone that I don't want that photo to go to.

By ‘private’, therefore, participants did not mean that information only be restricted to themselves. They enjoyed sharing information within chosen online networks, but key to their sense of privacy was control over the online content which involved them. This extended to concern over information posted about them online without their prior consent and the accessing of information they had posted by those who were not its intended audience.

## Getting to ‘know the other’

Establishing contact online is an example of where risk and opportunity are entwined: getting to ‘know the other’ online extends the possibility of meaningful relationships beyond physical boundaries but opens up the possibility of false presentation by ‘the other’, to which young people seem particularly susceptible (May-Chahal *et al*., 2012). The EU Kids Online survey (Livingstone *et al*., 2011) of nine-to-sixteen-year-olds distinguishes between young people establishing contacts online—which 30 per cent of young people had done—and the riskier act of meeting up with an online contact offline, which only 9 per cent had done, often without parental knowledge. In this study, while all participants had some Facebook Friends they had not met offline, the four participants making significant new relationships online were adult care leavers.

Three ways of meeting online contacts were described—first meeting people briefly offline before accepting them as a Facebook Friend, where the relationship deepened. The second way, through gaming, was described by Harry. While five participants participated in online games involving interaction with others, the interaction was largely minimal. Harry, though, took part in the online virtual world *Second Life* and described how interaction there could lead to establishing close friendships:

… you might just see someone's conversation randomly and you just jump in a little and say I like that and then … you will talk to them a bit more when you are online and you will build stronger relationships with them and stuff each time you talk to them, and then after a while of getting to know each other, you know, there'll be the thing with do you want to swap Facebooks and stuff and get to know each other a bit more … I have just made really strong relationships with them and stuff, so as they were a friend I know in person.

While only a small number of those Harry met in *Second Life* became Facebook Friends, in these cases, an absence of face-to-face contact was not a barrier to meaningful friendship. His description of the process of getting to know these friends had similarities with the process of getting to know someone offline but there was no intention, or seeming desire, to meet these people in person.

The final way of establishing online contacts was in accepting or making Friends requests to ‘Friends of Friends’ on Facebook who were not known offline. Graham reported having a girlfriend for the past month whom he had met in this way. Though she lived locally, their relationship had been conducted entirely online:

I messaged her saying ‘do you want to go out with me, blah, blah, blah’. She said ‘I'll have to think about it—I am not too sure’, and then a couple of days later she said ‘I will go out with you’.

Although Graham's intention was that the relationship would continue offline in the future, it was notable that he described himself as ‘going out’ with someone he had never physically met and that, when asked whether he had ever spoken to his girlfriend, he responded: ‘No, we have spoken on Facebook and MSN.’ This resonated with a Pew internet study (Lenhart *et al*., 2008) which found young people may conceive of forms of contact like texting and online communication as conversations rather than writing. It suggests the distinction between different synchronous and asynchronous digital communication highlighted by LaMendola (2010) may be of less significance to young people brought up with texting and online messaging as means of communication.

Graham did not voice any thoughts about the potential danger of meeting with someone he had only communicated with online. For Tracey, the fact she was an adult was a key difference underpinning her choice to make contacts online:

It's risky for everybody but you're more likely to protect yourself more when you're an adult than when you're a child.

The potential danger of meeting up with offline contacts was, however, underlined by an experience before Tracey reached adulthood. Although she did not wish to give further detail, she recounted meeting up with an online contact offline who turned out to be ‘somebody else’ and described it as a negative encounter. This was the only example given where meeting a contact made online resulted in difficulties.

By contrast, the most common, and marked, negative experience was some form of online verbal abuse by those known to participants offline. Six young people referred to occasions when they, or close friends, had experienced derogatory comments being made about them online or via text:

Diane: Sometimes you can get picked on, they [young people at school] use the Internet for stuff to bully people because they are not brave enough to go and say it their faces.

Int: So has that happened to people that you know?

D: Yes

Int: So what kind of stuff happens when they bully people?

D: They say stuff that's not true about them and they make some rumour up about them and make web pages up about them.

Int: So it's like publicly displaying it. So has that been resolved, how does a young person respond to that if that happens to them?

D: They mark it then go talk to teacher. They got that site too.

There was some suggestion that the experience of online verbal abuse was gendered in that all four female participants mentioned it as an issue, and one indicated this consisted of misogynist language. The potential overlap between offline and online vulnerability was also suggested by the fact that the participant who was most distressed by this experience was a young woman with a learning disability. However, the experience of online verbal abuse was not exclusive to young women and their views of social media were not shaped by these negative incidents. As Diane remarked about going online:

I feel in control every time. If I ever had any problems I would just tell my foster mum.

## The limitations of online connection

Participants' description of their relationships with their core virtual networks provided little to support Bauman's (2003) claim that human connections become shallower due to the rise of virtual proximity, and yet Bauman's (2003) description of connectivity for its own sake resonated with parts of young people's accounts. At school, Geoff responded to status updates on his mobile approximately every ten minutes, including during lessons when he might have the phone confiscated. When asked why, he responded ‘Why not, just cos?’. Diane complained of the trivial nature of some of her friends' status updates yet felt the need to respond to them quickly for fear that ‘they would fall out with me … [b]ecause they're impatient’. Nick described that his mobile's audible push alerts, when one of his online Friends posted, could awaken him at night, but he decided not to change the settings:

Because it's easier, because that way if someone has been on at night while I have been sleeping, it gives me something, it makes you more active, doesn't it, you're reading something and you are sat up?

These accounts resonate with Livingstone's (2008) claim that young people confirm their position in friendship networks by regular online posting. They also provide some support to Bauman's observation regarding the show of connection, with the greatest fears being those ‘of being caught napping, of failing to catch up with fast moving events, of being left behind’ (Bauman, 2005, p. 2).

Participants were, however, keen to note that online connection was not the sum total of their social interaction and contrasted time spent online with social activities offline. Geoff emphasised that he used Facebook ‘at night after I've already been out’ while engaging in physical activities, usually with others (‘swimming’, ‘riding a bike’, ‘bowling’, ‘going to the park’) and practical activities such as household tasks and ‘sorting out my current situation’ were described, positively, as alternatives to using social media. Underlying this distinction was the sense that young people themselves felt that online interaction, although valued and enjoyable, had its limitations and needed to be balanced by offline activity.

## Conclusion

Current evidence suggests some groups of young people are more vulnerable to the dangers connected to digital media use. In this study, the risks of meeting online contacts offline were highlighted by Tracey, the majority of participants had received some form of online verbal abuse from other young people they knew and two care leavers' accounts suggested potential excessive internet use. There was also a suggestion that female participants may experience greater difficulty in respect of online verbal abuse.

Notably, however, these experiences were not markedly more negative than wider peer experience revealed in other research. Participants were also accessing the internet and mobiles as regularly, their social networks appeared of broadly comparable size and their primary interactions were with those they already knew and communicated with offline. A situation of bounded agency applied whereby, despite familial and social differences between this group of participants and their peer group, they were still using digital media in ways that made sense to their own ‘reflexive life projects’ (Furlong, 2009, p. 353).

This is not an argument for complacency. However, it suggests the importance of a nuanced approach which does not assume the use of new technology by looked after children and care leavers to be inherently problematic or to pose qualitatively different challenges. While digital media played a central part in participants' social lives, the underlying issues of friendship, chat, group membership and group exclusion appear similar to those which marked relationships in a pre-digital age. The solidity of social relationships—for good and bad—had not melted away as fundamentally as some accounts have claimed.

The data also provide little evidence that these care-experienced young people were using new technology in ways which might significantly enlarge social networks. Participants' use of digital media revolved around a fairly narrow range of activities—primarily communication via social networking sites and texting to people they already knew offline. This provided useful and valued, if limited and individualised, sources of social support. In a small number of cases, friendships were forged online, but these were the exception, and restricted to care leavers. While this finding is again consistent with peer group usage (see Livingstone *et al*., 2011), it does suggest there is space for greater awareness of digital literacies which can support creative interaction using digital media, as highlighted by Guzzetti (2006).

That care leavers experienced greater barriers to accessing the newest technology, and some greater difficulty getting online, highlights the need to think through access to digital media at important transition points for looked after children, such as when returning to parental care or leaving care, as some social support and friendships could be lost through a lack of connectivity. The importance of exploring young people's preferences regarding digital media use is also emphasised. Goodyer (2011, p. 156) reasonably suggests social workers working with children in care should consider using platforms such as ‘e-mail or texting or new ones like tweeting, blogging and social networking sites’ to keep in regular contact with them. However, young people in this study only consistently named texting as a preferred means of professional contact. E-mail, Twitter and blogs were not widely used, while some young people in the study would have likely been wary of opening up their Facebook profiles to adult professionals.

Three priority areas for future research are suggested. First, there is a body of data on a broad range of issues regarding young people's use of technology through the EU Kids Online studies (see Livingstone *et al*., 2011). A comparative study with a representative sample of looked after children would help establish more clearly whether there are substantive differences and, if so, which particular groups of looked after children and care leavers are in need of greatest support regarding their use of new technology. A second avenue is ethnographic research which seeks to explore the digital practices of looked after children or care leavers. It would be particularly valuable for such study to include young people who may be deemed particularly ‘vulnerable’, such as looked after children with a disability or young people in care or leaving care whose personal circumstances appear to put them at greater risk. Observing the digital practices of looked after children in important stages of transition—those entering care, returning to parental care or transitioning to adulthood – would also be valuable. Finally, little is currently known about how, or whether, professionals are including consideration of digital media in their work with looked after children and care leavers: research which explores their understanding, attitudes and practice towards the use of digital media by the young people they work with and which helps identify professional needs for development would also be of merit.
